# Fluid overload is an independent risk factor for acute kidney injury in critically Ill patients: results of a cohort study

**DOI:** 10.1186/s12882-017-0460-6

**Published:** 2017-02-01

**Authors:** Nawal Salahuddin, Mustafa Sammani, Ammar Hamdan, Mini Joseph, Yasir Al-Nemary, Rawan Alquaiz, Ranim Dahli, Khalid Maghrabi

**Affiliations:** 1 Adult Critical Care Medicine, King Faisal Specialist Hospital & Research Center Riyadh, Riyadh, Saudi Arabia; 2 Critical Care, Department of Nursing, King Faisal Specialist Hospital & Research Centre Riyadh, Riyadh, Saudi Arabia; 3 Department of Surgery, King Faisal Specialist Hospital & Research Centre Riyadh, Riyadh, Saudi Arabia

**Keywords:** Acute kidney injury, Fluid overload, Renal congestion, Chloride-liberal fluids, Critical illness

## Abstract

**Background:**

Acute Kidney injury (AKI) is common and increases mortality in the intensive care unit (ICU). We carried out this study to explore whether fluid overload is an independent risk factor for AKI.

**Methods:**

Single-center prospective, observational study. Consecutively admitted, ICU patients were followed for development of AKI. Intravenous fluid volumes, daily fluid balances were measured, hourly urine volumes, daily creatinine levels were recorded.

**Results:**

Three hundred thirty nine patients were included; AKI developed in 141 (41.6%) patients; RISK in 27 (8%) patients; INJURY in 25 (7%); FAILURE in 89 (26%) by the RIFLE criteria. Fluid balance was significantly higher in patients with AKI; 1755 ± 2189 v/s 924 ± 1846 ml, *p* < 0.001 on ICU day 1. On multivariate regression analysis, a net fluid balance in first 24 h of ICU admission, OR 1.02 (95% CI 1.01,1.03 *p* = 0.003), percentage of fluid accumulation adjusted for body weight OR1.009 (95% CI 1.001,1.017, *p* = 0.02), fluid balance in first 24 h of ICU admission with serum creatinine adjusted for fluid balance, OR 1.024 (95% CI 1.012,1,035, *p* = 0.005), Age, OR 1.02 95% CI 1.01,1.03, *p* < 0.001, CHF, OR 3.1 (95% CI 1.16,8.32, *p* = 0.023), vasopressor requirement on ICU day one, OR 1.9 (95% CI 1.13,3.19, *p* = 0.014) and Colistin OR 2.3 (95% CI 1.3, 4.02, *p* < 0.001) were significant predictors of AKI. There was no significant association between fluid type; Chloride-liberal, Chloride-restrictive, and AKI.

**Conclusions:**

Fluid overload is an independent risk factor for AKI.

## Background

Acute Kidney injury (AKI) develops in 55–66% of critically ill patients [1, 2] with an attributable mortality of 15–80%, dependent on the severity of renal dysfunction [3, 4]. The ICU physician has no influence over the usually described AKI risk factors; severity of illness, hemodynamic instability, comorbid illnesses, heart failure, cirrhosis and emergency surgery [2, 5–7], however if potentially modifiable factors can be identified there is a real potential to reduce either the incidence or the severity of any renal injury.

Fluid overload is related to an increase in overall mortality with critical illness [8, 9]. In patients with established AKI, further fluid overloading has been linked with lower survival and lesser renal recovery [10–14].

There is emerging evidence that suggest a role for fluid overlcoad as a causative factor for AKI [15, 16]. It is biologically plausible that volume loading leads to organ congestion and a resultant decrease in renal blood flow [17, 18]. Whether fluid overload leads to cellular dysfunction from reduced perfusion, tissue hypoxia or by direct disruptions in cellular function is still not clear. Interestingly the renal capsule itself may cause a ‘compartment syndrome’ by its inability to distend when renal congestion occurs. Previous investigators have shown that a capsule ‘ectomy’ improves renal blood flow [19]. An additional risk factor for AKI appears to be chloride—liberal intravenous fluids that contain ‘supra-physiological’ concentrations of chloride, such as 0.9% saline, a routine intravenous solution in most intensive care units (ICUs) [20–22]. Recent work has linked chloride—liberal intravenous solutions with AKI [23, 24].

The objectives of this study were to determine whether the potentially modifiable risk factors of Fluid overload and Fluid Type (chloride—liberal versus chloride—restrictive) are risk factors for AKI in critically ill patients. We hypothesized that patients admitted to the ICU may be at a higher risk of AKI if they had a net positive fluid balance or received chloride—liberal fluids for both resuscitation and as maintenance therapy.

## Methods

This study is reported following the STROBE statement checklist for observational studies [25].

### Study design and setting

This was a prospective, cohort study carried out on consecutive, critically ill, adults admitted to Medical and Surgical ICUs at a tertiary care, referral hospital over a 5-month period between 8/2013 and 12/2013.

### Operational definitions

Acute Kidney Injury (AKI) was defined according to the RIFLE Classification [26] of renal dysfunction and categorized into RISK, INJURY and FAILURE. Renal dysfunction was defined using both increases in creatinine from admission values and urine output measured as urine volume in milliliters/patient’s baseline weight in kilograms/h; as weights are routinely measured on admission for all ICU admissions, Serum creatinine values were measured at admission and daily for up to the seventh ICU day. Serum creatinine from the patients’ outpatient records provided a baseline creatinine value and values were available for all patients. Serum creatinine was measured using the COBAS Integra Creatinine plus ver. 2 Assay, Roche Diagnostics Corporation. This is an enzymatic method based on the determination of hydrogen peroxide after conversion of creatinine with the aid of creatininase, creatinase, and sarcosine oxidase. Patients were screened for the development of post-admission AKI on a daily basis. Pre-Existing Renal Impairment was defined as elevated serum creatinine in the pre-ICU admission medical records, i.e: abnormal baseline creatinine. Fluid Overload was defined as a net positive fluid balance. Additionally we computed the percentage of fluid accumulation adjusted for body weight by using the formula; Total intake (liters) – Total output (liters)/baseline body weight and expressed as a percentage [27]. Intravenous fluids were classified into ‘Chloride-liberal’ fluids, i.e.: those containing supraphysiological concentrations of chloride (0.9% saline, 20 and 5% albumin) and ‘Chloride-restrictive’, which contain chloride concentrations closer to plasma (0.45% saline, Ringer’s lactate).

### Participants

Consecutive, critically ill adults admitted within the pre-specified study period were included. Routine, postoperative patients admitted for less than 72 h, patients with AKI at ICU admission and end-stage renal disease patients treated by chronic dialysis were excluded. Crystalloids used in the study patients were; Lactated Ringers (sodium chloride, potassium chloride, sodium lactate and calcium chloride) injection, 0.9% Sodium Chloride Injection, USP, 0.45% Sodium Chloride Injection, USP.; manufactured by Baxter,Baxter Healthcare Corporation, DeerfieldIL60015 USA. Colloids used were; Human Albumin 5 and 20% manufactured by Biotest Pharma GmbH, Landsteinerstraße 5, 63303 Dreieich, Germany.

### Variables

The primary outcome variable was the development of AKI as defined above*.* Other recorded variables were, 24-h fluid balance, types of intravenous fluids received during the ICU stay, comorbidities, demographics (age, gender, operative status), severity of Illness scores (APACHE II [28],SAPS II [29]), routine hematological, biochemical and organ dysfunction/physiological (vasopressors, renal replacement therapy (RRT), mechanical ventilation) data, usage of potentially nephrotoxic medications such as NSAIDs, iodinated contrast, intravenous starch, vancomycin, Colistin.

### Statistical analysis

Continuous data was tested for normality; measures of central tendency were compared as means ± standard deviations (SD) using the Student *t* test for normally distributed variables and as medians (interquartile range, IQR) using the Mann–Whitney *U* test for skewed data. Categorical variables were compared using the CHI^2^ test or the Fisher Exact test for *n* < 5. Fluid volumes were dealt with as continuous variables whilst fluid types were classified into either ‘Chloride-liberal’ or ‘Chloride-restrictive’ and correspondingly dealt with as continuous variables. Logistic regression analysis was performed to determine the predictive ability of variables for AKI. Univariate and multivariate techniques were used, and for multivariate regression, a backward mode with a threshold 0.10 was used for elimination. Multivariate associations were reported as odds ratios (OR) with 95% confidence intervals. A sensitivity analysis was carried out with the serum creatinine adjusted by fluid balance, using the formula;

$$ Adjusted\  Serum\  Creatinine = Serum\  Creatinine\  x\  correction\  factor $$

when correction factor = 1 + {cumulative fluid balance (L)/(admission body weight (kg) × 0.6)} [30]. A two-sided *p* value of < 0.05 was considered as statistically significant. All analyses were carried out using IBM SPSS version 22.0.

## Results

### Participants and descriptive data

Three hundred and thirty-nine patients were included; AKI developed in 141 (41.6%). Mean time to development of AKI was 1.9 ± 0.4 days (range 1,3). Mean age was 51 ± 20.4 years, 167 (49%) patients were male. Mean APACHE II score was 22 ± 12.8 and SAPS II score was 35.4 ± 18.9. Severe sepsis/septic shock was the admitting diagnosis in 129 (38%) patients, 56 (16.5%) were admitted with respiratory failure and 60 (18%) patients were post-operative.

**Fig. 1:**
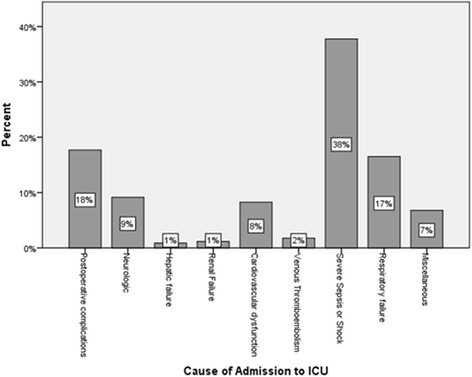
Causes of ICU admission

Comorbid conditions included; malignancy 149 (44%), diabetes mellitus 110 (32%), chronic liver disease 81(24%), connective tissue disease 81 (24%) and congestive heart failure (CHF) 23(7%). During ICU admission, patients received vancomycin, 223 (66%), Colistin 97 (29%), aminoglycosides 57 (17%), NSAIDs 0 (0%), iodinated contrast 73 (21.5%). Thirty- three (10%) patients had pre-existing renal impairment. Net fluid balance at 24 h of ICU admission was +1027 ml (IQR 25% 0, 75%, +1710). Loop and thiazide diuretics were used in 238 (70%) patients (Fig. 1).

**Fig. 2:**
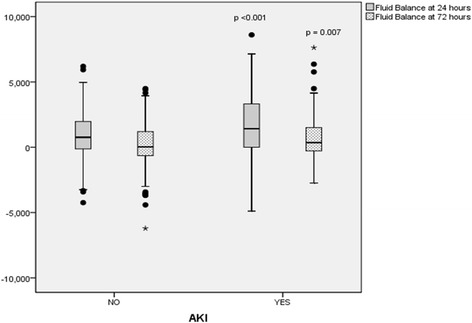
Comparison of Fluid balances in patients with and without AKI. Significant differences between fluid balance at 24 h of ICU admission, *p* < 0.001 and at 72 h, *p* = 0.007

In patients who developed AKI, mean arterial pressures were significantly lower; 66.8 ± 18.6 v/s 71 ± 13.7, *p* = 0.015 on Day 0 and 67.8 ± 18 v/s 73.5 ± 16, *p* = 0.002 on Day 3, with significantly higher serum lactate levels, 2.7 ± 1.9 v/s 2.0 ± 2.6, *p* = 0.02. No significant differences were found in lactic acid clearance; serum lactate at 6 h 1.6 (2) v/s 1.9 (2), p value NS or central venous oxygen saturation (ScvO2); 68.5 ± 24.8 v/s 71.5 ± 22.6, p value NS at admission and 67 ± 25.4 v/s 65.7 ± 25, p NS at 24 h. Fluid balance was significantly higher in patients with AKI; 1755 ± 2189 v/s 924 ± 1846 ml, *p* < 0.001 on ICU day 1 and 665 ± 1686 v/s 167 ± 1658 ml, *p* = 0.007 on day 3 (Fig. 2). There was no significant differences found in the volumes of fluid types received for both resuscitation or for maintenance; Chloride-Liberal fluids in first 24 h 4880 ml (IQR 3342) v/s 5500 (IQR 4729), p 0.20, Chloride-Restrictive fluids in first 24 h 3665 (2000) v/s 2610 (IQR 2000), p 0.22, Chloride-Liberal fluids in first 48 h 1079 (IQR 894) v/s 970 (IQR 838), p 0.33 and Chloride-Restrictive fluids in first 48 h 1949 (IQR 650) v/s 1983 (IQR 763), p 0.32.

Serum chloride values also were not different between the AKI and non-AKI group throughout the ICU stay, 112 ± 21 v/s 108 ± 28 mmol/L, *p* = 0.22 (Table 1).

**Table 1:** Comparison of variables in critically ill patients with and without Acute Kidney Injury

	AKI	No AKI	*p* value
	*N* = 141 (41.6%)	*N* = 196 (56%)	
Age	56 ± 19	47 ± 20.5	<0.001
Male	76 (51%)	91 (48%)	0.57
APACHE II	25 ± 12	21 ± 13.5	0.006
SAPS II	42 ± 20	30 ± 16	<0.001
Post-operative	40 (27%)	68 (35.6%)	0.09
CHF	16 (11%)	7 (4%)	0.009
Diabetes	52 (35%)	58 (30%)	0.54
COPD	5	6	0.93
Malignancy	65	84	0.86
Cirrhosis	39	42	0.44
Renal Impairment prior to ICU admission	15	18	0.91
Connective Tissue Disease	36	45	0.85
*Cause of ICU Admission*			0.31
Severe Sepsis/Septic Shock	55	73	
Postoperative Complications	24	36	
Respiratory Failure	20	36	
Acute Neurologic event	12	19	
Heart Failure/Arrhythmia	17	11	
Others^a^	15	21	
Vancomycin	106 (72%)	117 (61%)	0.046
Colistin	48 (32%)	49 (26%)	0.10
Aminoglycosides	22 (15%)	35 (18%)	0.29
*Serum Creatinine (mmol/L)*			
at admission	145 ± 116	86.5 ± 168	<0.001
Day 1	153.6 ± 117	75.6 ± 17	< 0.001
Day 2	148.4 ± 107	73.1 ± 7	< 0.001
Day 3	147.3 ± 101	69.2 ± 6	< 0.001
*Urine output (ml/kg/hour)*			
Day 1	0.62 ± 0.6	1.36 ± 0.9	< 0.001
Day 2	0.74 ± 0.8	1.49 ± 0.7	< 0.001
Day 3	0.71 ± 0.8	1.43 ± 1	< 0.001
MAP (mmHg) mean (SD)			
Day 0	66 ± 16	71 ± 14	0.033
Day 1	68.6 ± 13.3	70.7 ± 11.8	0.12
Day 2	69.2 ± 15.8	71.4 ± 15	0.20
Day 3	67 ± 18	74 ± 15	0.002
*Patients on Vasopressors*			
Day 1	76	62	<0.001
Day 2	77	65	0.001
Day 3	71	58	0.001
*Serum Lactate (*mmol/L)			
at admission	2.7 ± 1.9	2.0 ± 2.6	0.02
at 6 h	1.6 (2)	1.9 (2)	0.91
*ScvO2*			
at admission	68.5 ± 24.8	71.5 ± 22.6	0.86
at 24 h	67 ± 25.4	65.7 ± 25	0.66
*Fluid Balance* (ml) median(IQR)			
Day 1	+ 1500 (3254)	+ 869 (2284)	0.002
Day 2	+ 744 (2292)	+ 486 (2250)	0.017
Day 3	+ 518 (1521)	0 (1604)	0.043
*Fluid Volume* (ml) median(IQR)			
Chloride-Liberal fluids in 1st 24 h	4880 (3342)	5500 (4729)	0.20
Chloride-Restrictive solutions 1st 24 h	3665 (2000)	2610 (2000)	0.22
Chloride-Liberal fluids by 48 h	1079 (894)	970 (838)	0.33
Chloride-Restrictive fluids by 48 h	1949 (650)	1983 (763)	0.32
Serum Chloride *(*mmol/L)			
at 24 h	111 ± 18.7	113 ± 17	0.69
at 48 h	113 ± 15.5	113 ± 15	0.17
ICU mortality	19	27	0.89
28 day Survival	114	159	0.74

^a^Includes Venous thromboembolism, decompensated cirrhosis

*Abbreviations*: *APACHE* Acute Physiology Age Chronic Health Evaluation, *SAPS* simplified acute physiology score, *MAP* mean arterial pressure, *CHF* congestive heart failure

### Outcomes

**Fig. 3:**
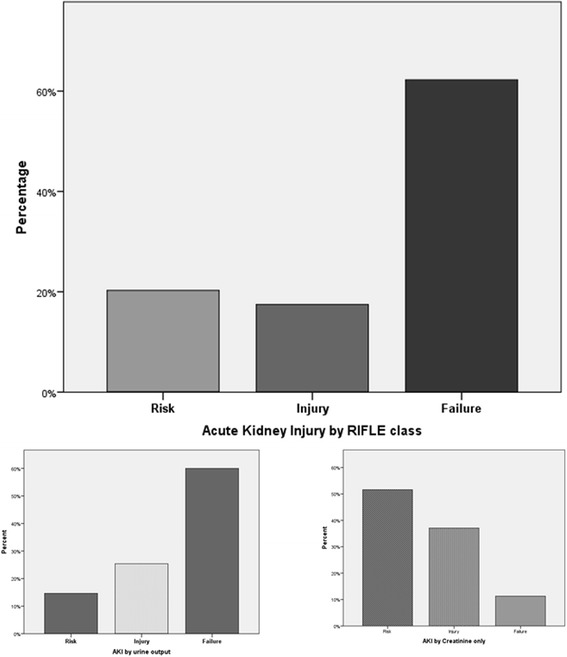
Incidence of AKI as defined by RIFLE Classification; both creatinine and oliguric criteria, by creatinine only and oliguria only [26]

AKI developed in 141 (41.6%) patients; RISK in 27 (8%) patients; INJURY in 25 (7%); FAILURE in 89 (26%) by the RIFLE classification and using both creatinine and urine output criteria; AKI defined only by increase in serum creatinine developed in 64 (19%), whilst AKI by urine output criteria developed in 130 (38.3%). When serum creatinine was adjusted for fluid balance, AKI was diagnosed in 153 (45%) patients (Fig. 3).

Ninety-three (27%) patients were started on continuous renal replacement therapy (CRRT). Mean ICU length of stay was 9.3 ± 11.3 days. ICU survival was 86% (292 patients), 28-day survival 80% (270 patients).

### Univariate outcome data

On univariate regression analysis; development of AKI was significantly associated with net fluid balance on the first ICU admission day, OR 1.2 (95% CI 1.1,1.3, *p* < 0.001); percentage of fluid accumulation adjusted for body weight OR1.01 (95% CI 1.004,1.018, *p* = 0.002); net fluid balance on the 3rd ICU admission day OR 1.02, 95% CI 1.01,1.03, *p* = 0.027; age, OR 1.02 (95% CI 1.01,1.03, *p* < 0.001); CHF, OR 3.1 (95% CI 1.2,7.9, *p* = 0.013); APACHE II scores, OR 1.02 (95% CI 1.0,1.04, *p* = 0.01); SAPS II scores, OR 1.02 (95% CI 1.007,1.03, *p* = 0.002); Admission MAP OR 0.98 (95% CI 0.96,0.99, *p* = 0.008); Vasopressors at admission, OR 2.5 (95% CI 1.5,4.0, *p* < 0.001) and for greater than 24 h, OR 2.37 (95% CI 1.48, 3.7, *p* < 0.001); admission lactate OR 1.17, 95% CI 1.03.1.33, *p* = 0.014 and Colistin, OR 2.52 (95% CI 1.52,4.18, *p* < 0.001). Fluid balance remained a significant predictor of AKI as defined by urine output only, OR 1.01 (95% CI 1.007,1.02, *p* = 0.002 and by creatinine only, OR 1.012 (95% CI 1.008,1.016, *p* = 0.013) and by serum creatinine adjusted for fluid balance OR 1.02 (95% CI 1.01,1.03, *p* < 0.001). There was no significant association between fluid types and AKI; Chloride-liberal, *p* = 0.20 and Chloride-restrictive, *p* = 0.22, serum chloride and AKI, *p* = 0.46 or ICU, *p* = 0.14 and 28-day mortalities, *p* = 0.81.

### Multivariate analysis

After adjusting for covariates, a net fluid balance in first 24 h of ICU admission, OR 1.02 (95% CI 1.01,1.03 *p* = 0.003), percentage of fluid accumulation adjusted for body weight OR1.009 (95% CI 1.001,1.017, *p* = 0.02), fluid balance in first 24 h of ICU admission with serum creatinine adjusted for fluid balance, OR 1.024 (95% CI 1.012,1,035, *p* = 0.005), Age, OR 1.02 95% CI 1.01,1.03, *p* < 0.001, CHF, OR 3.1 (95% CI 1.16,8.32, *p* = 0.023), vasopressor requirement on ICU day one, OR 1.9 (95% CI 1.13,3.19, *p* = 0.014) and Colistin OR 2.3 (95% CI 1.3, 4.02, *p* < 0.001) remained significant predictors of AKI (Table 2).

**Table 2:** Predictors of acute kidney injury by regression analysis

Variable	Exp (B)	95% CI	*p* value
*Univariate analysis*			
Net fluid balance on the first ICU admission day	1.2	1.1,1.3	<0.001
Net fluid balance on the 2nd ICU admission day	1.02	1.01,1.03	0.11
Net fluid balance on the 3rd ICU admission day	1.02	1.01,1.03	0.027
Percentage of fluid accumulation adjusted for body weight	1.01	1.004,1.018	0.002
Age	1.02	1.01, 1.03	<0.001
CHF	3.1	1.2, 7.9	0.013
APACHE II scores	1.02	1.01, 1.04	0.011
SAPS II scores	1.02	1.007, 1.03	0.002
Admission MAP	0.98	0.96, 0.99	0.008
Vasopressors at admission	2.5	1.5, 4.0	<0.001
Vasopressors at 24 h	2.37	1.48, 3.7	<0.001
Admission serum lactate	1.17	1.03, 1.33	0.014
Colistin	2.52	1.52, 4.18	<0.001
*Multivariate analysis*			
Net fluid balance on the first ICU admission day	1.02	1.01,1.03	0.003
Percentage of fluid accumulation adjusted for body weight	1.009	1.001,1.017	0.02
Vasopressors at 24 h	1.9	1.13,3.19	0.014
Age	1.02	1.01,1.03	0.001
Congestive Heart Failure	3.1	1.16,8.32	0.023
Colistin	2.3	1.3, 4.02	<0.001

## Discussion

The main findings of this study are that a positive fluid balance in the first 24 h of ICU admission is associated with a significant risk of AKI in a mixed critically ill population. We were not able to determine an association between types of intravenous fluids and AKI.

Fluid overload that occurs either with resuscitation or with indiscriminate use is now becoming recognized as a risk factor in itself for ICU complications. In 2011, Liu et al. [15] published a posthoc analysis of the Fluid and Catheter Treatment Trial (FACTT) data where they described a higher incidence of AKI after adjustment for fluid balance in patients allocated to the fluid liberal group (66% v/s 58%, *p* = 0.007). Hassinger et al. [16] in an observational study of 98 pediatric post-cardiac surgery patients described greater risk of AKI with postoperative fluid overload (described as a fluid balance 5% above body weight). Cumulative fluid administered was an excellent predictor of pediatric-modified Risk, Injury, Failure, Loss and Endstage (AUC 0.963, 95% CI 0.91-1.0, *P* = 0.002). Wang et al in the recently published Beijing Acute Kidney Injury Trial [27] conducted in 30 ICUs and that included 1172 patients with AKI, showed that fluid overload was an independent risk factor for the incidence of AKI (odds ratio 4.508, 95% confidence interval 2.900 to 7.008, *p* < 0.001) and for increased severity of AKI. AKI patients who died had a higher cumulative fluid balance during the first 3 days (2.77 [0.86–5.01] L versus 0.93 [−0.80 to 2.93] L, *p* < 0.001) compared to survivors.

Fluid overload is postulated to cause renal dysfunction in a number of ways. Renal congestion and interstitial edema lead to distortion of the renal architecture and impaired metabolite diffusion, compromised tissue oxygenation. Capillary and lymphatic obstruction ensues with further organ congestion [17, 18]. The renal capsule limits the kidney’s ability to accommodate increasing hydrostatic interstitial pressures and eventually leads to reduce renal perfusion and glomerular filtration. Decapsulation, in an animal model of resuscitation, has been described to protect the kidney from injury Increased intra-abdominal pressures from fluid loading compromise renal blood flow and contribute further to fluid overload by reduced salt and water excretion [19].

An association between fluid overload and renal dysfunction however does not prove causality. It is possible that fluid overload represents an epi-phenomenon and is a reflection of the severity of hypoperfusion and the resuscitative response. Our findings are limited by our observational design and the observed associations may be subject to bias from selection, confounding or random error. We attempted to control for confounders by using regression analysis. Another limitation is the external validity or generalizability of our results to other critically ill patients since we collected data only from a single institution. Possible biases are also parameters followed for fluid administration and blood transfusion. Currently our ICU protocols attempt goal-directed fluid resuscitation using inferior vena cava ultrasound measurements to guide fluid administration. Blood transfusion triggers are serum hemoglobin values < 7 mg/dl. Since these are universally accepted parameters by most ICU physicians, we believe that the fluid administration and transfusion practices at our institution may cause limited bias.

We did not find any association between the development of AKI and whether predominantly chloride-liberal or chloride-restrictive fluids were used for either initial resuscitation or subsequent maintenance. These findings are contrary to recent work published by Yunos et al. [23], in a pre and post-intervention study on 1530 critically ill patients found that a chloride-restrictive fluid strategy resulted in a significant reduction in AKI, need for renal replacement therapy and increase in creatinine as compared to a control group given chloride-liberal fluids. Similarly we recently reported on a significant association between AKI and larger volumes of chloride-liberal (hyperchloremic) fluids in 158 post-liver transplant patients [24]. In that study the AKI-group had significantly higher serum chloride levels compared to transplant recipients that did not develop AKI. In our current study with a cohort of mixed population of critically ill patients, the mean serum chloride levels throughout the ICU stay were not significantly different. Therefore, it is possible that the AKI seen in patients receiving chloride-liberal fluids is due to chloride toxicity and occurs when relative hyperchloremia develops.

Animal studies have demonstrated renal vasoconstriction and thromboxane release after chloride infusions [21] with chloride infusions increasing delivery to the macula densa that stimulates glomerulotubular feedback leading to afferent arteriole constriction, mesangial contraction and resultant decreases in GFR [22]. Clearly, further work to establish a cutoff hyperchloremic value can be considered.

In this study, we also found no significant association between a diagnosis of sepsis and AKI; however, significant associations with baseline mean arterial pressures and vasopressor usage and AKI suggest that the renal impact of sepsis maybe more a function of hemodynamic instability. In addition, there was no mortality difference between the AKI and non-AKI cohorts. Possibilities are the beneficial impacts of early and adequate hemodynamic resuscitation (as evidenced by the normalization of serum lactate in both groups by 6 h or the mixed venous saturation at admission) or the benefits of early renal replacement therapy (CRRT was started in all RIFLE 3 within the first 24 h).

## Conclusions

In summary, large infusions of fluids may predict a higher risk of AKI in critically ill patients. Our findings support the hypothesis that ‘routine’ intravenous fluids may not be routine and in themselves be associated with organ dysfunction. Our results can be used to build hypotheses for further controlled trials.

## Abbreviations

AKI: Acute kidney injury
APACHE II: Acute physiologic and chronic health evaluation II
GFR: Glomerular filtration rate
OR: Odds ratio
RIFLE: Risk injury failure loss end-stage renal failure
RRT: Renal replacement therapy
SAPS II: Simplified acute physiology score
